# Analysis of the Influence of Blanking Clearance on the Wear of the Punch, the Change of the Burr Size and the Geometry of the Hook Blanked in the Hardened Steel Sheet

**DOI:** 10.3390/ma12081261

**Published:** 2019-04-17

**Authors:** Jacek Mucha, Jacek Tutak

**Affiliations:** 1 The Faculty of Mechanical Engineering and Aeronautics, Department of Mechanical Engineering, Rzeszów University of Technology, Al. Powstańców Warszawy 8, 35-959 Rzeszów, Poland; 2 The Faculty of Mechanical Engineering and Aeronautics, Department of Applied Mechanics and Robotics, Rzeszów University of Technology, Al. Powstańców Warszawy 8, 35-959 Rzeszów, Poland; tutak.j@prz.edu.pl

**Keywords:** blanking, burr height, sloping face of the punch, damage of the punch, sheet C45

## Abstract

In this thesis, the results of an experimental analysis of blanking angled hooks with a punch of a sloping face in a thin steel sheet with a hardness of 55 HRC are presented. The blanking punch was made of K340 cold-work tool steel. Tests were carried out for three values of clearance, 5%, 10%, and 15% of sheet thickness. The results of the analysis of the influence of the number of cuts made by the punch on the growth of the burr at the sheet edge were presented. Moreover, the influences of the clearance on the initial values of burr (*b_h_*), deflection (*H_b_*), and the bending radius (*R_b_*) of the hook have been shown. The influence of the friction path on the intensity of degradation of working surfaces and the blanking edges of the punch was also demonstrated. The obtained results allow the selection of the proper clearance and new tool materials for blanking blades working in particularly difficult tribological conditions.

## 1. Introduction

During blanking, due to the pressure on the blanking edge of the punch and the die, a complex stress distribution is achieved. After plasticizing the material, depending on the method of the process, different geometry of the intersection surface and burr size can be obtained. 

**Figure 1:**
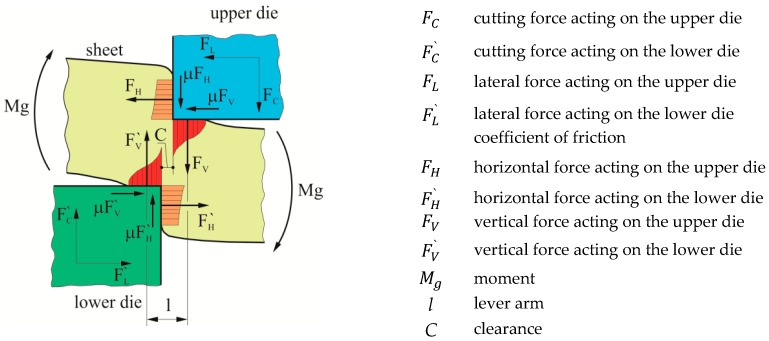
The system of forces and pressures during punching.

In the case of deformation of the sheet material, the punching force causes the tools to be loaded. The greatest pressures are close to the tool’s cutting edges. The pressure of the punched material on the punch surface and the friction causes intensive tool wear [1]. The vector of reaction forces for the tools is inversely directed to the working movement of the punch. This causes a bending of the sheet, which results in the lateral action of forces. The clearance between the tool edges determines the value and orientation of the contact force. The model of reaction forces and the bending moment is shown in Figure 1 [2].

The blanking process is widely used to manufacture sheet metal parts from different materials. The cut elements are usually subjected to further plastic working, but there are also those that are directly used in the assembly process of complex products. The blanking of the elements with a punch of flat surface has been very well presented in a number of works [3,4,5,6,7,8,9,10,11,12,13,14,15,16,17]. When punching with a flat face, the edge pressure on the sheet is uniform. For punch with a sloping face, there is a gradual blanking of the sheet material [18]. The location of the punching force on the sheet material changes. As the punch penetrates into the sheet, the material separation moves along the blanking edges. Blanking with the inclined blanking edge allows the lowering of the maximum pressure force [18]. The angle of inclination of the face of the punch also influences the amount of deformation of the stamped element [19]. Gürün and others [19], showed that blanking axisymmetric elements with a punch with an inclined surface causes deformation of the shape in two perpendicular surfaces. They presented the influence of the angle of inclination of the punch face on the maximum punching force. They showed, in the case of punching DC01 sheet material with a thickness of 1 mm, that for the greater angle of punch face inclination the maximum force and total work is lower. However, a larger stroke of the punch is necessary for blanking. In addition, it has been shown that a larger angle of inclination of the stamp’s face causes greater deformation of the shape of the final product.

The use of punches with a sloping face results in a reduction of the punching force. However, too large an angle can cause large burr [3]. Lin [20], presents the results of blanking with a punch with two faces inclined at the angle of 12°. The tests were carried out using a 0.77 mm thick sheet metal. They analyzed the influence of clearance on the punch wear.

The intensity of the burr height increase depends on the speed of wear of blanking tool edges [4,5,6]. In the case of blanking thin sheets with special magnetic properties, it is important to choose the appropriate clearance, so the area of the hardened material will be the smallest [4]. Tool wear is particularly important when blanking thin sheets. The quality of the intersection surface in the blanking process is influenced by the type of punch material [5,6]. Works [4,5,6] present the influence of the punch material on its wear (tungsten carbide, M2, M3: 2). Tool wear depends on the size of the clearance, punch material, and sheet material [7,8,9,10,21,22]. When blanking hard materials, the problem is to obtain the right quality of cut surface. Fazily and other authors [23], presented an analysis of the blanking of an element from AZ31B magnesium alloy. As a result of heating the punched material, they increased the smooth blanking zone and significantly reduced the punching force. In the case of steel sheets with high hardness obtained after heat treatment, the punching clearance should be considerably higher than for soft materials [24]. When blanking very hard materials, very high pressures occur on the face (at the blanking edge). Such a cyclically loaded punch is more worn out on the face side [24]. Increase of the tool wear resistance can be achieved by applying hard coatings. In [21], the authors have outlined the effect of the anti-wear coating on the friction and wear conditions of tool materials. In [7], the authors presented the issues of the influence of heat treatment and the hardness of punches on the course of change in the quality of the cut surface. The higher the hardness of the America Iron and Steel Institute (AISI) D2 steel punches was, the lower the punch wear and the change in the quality of the cut surface were. Blanking asymmetrical outline elements is more complex than blanking circular elements. Subramonian [8] presented the influence of the size of the radius of the cut profile on the wear of the blanking edge. Blanking tests were carried out for 0.25 mm thin sheet metal and several radius values: *R* = 0.15, 0.25, 0.5, and 1 mm. He characterized the influence of the radius/sheet thickness ratio on the normalized stress value. The work is important from the point of view of thin-element blanking. Guo [9] conducted a circular punch blanking test with a 0.15 mm diameter and 0.2 mm thick sheet metal. In the case of blanking round elements, their size, thus the punch diameter, influenced the punch wear. The influence of the SKD-11 steel punch diameter on the quality of die-cut sheet metal parts was described by Lawanwong [10]. He also showed how using regenerated punches affects the quality of elements during blanking.

Often, in the production processes of sheet metal parts, flange trimming is applied after the pressing process. Appropriate direction of the sheet surface inclination to the punch results in an improvement of the quality of the intersection surface [25]. Golovashchenko [26], presented the influence of the method of supporting the cut sheet from the aluminum alloy AA6111-T4 and the clearance on the geometric quality of the intersection surface and on the formation of burr. The shearing was made for an aluminum alloy sheet and a flat-faced cutter. In the next paper [27] he presented a modified shearing process in the case of using an inclined sheet surface to the face of the cutter. He showed that the inclination angle of the sheet can be chosen with a relatively large clearance so that a flat surface of the intersection without burrs will be obtained. The mechanism of separation of materials during shearing can be changed by modifying tool arrangement [28]. In paper [29], the authors presented the results for the DP500 high-strength sheet shearing tests using an additional element supporting the sheet material. Separation of material with an open cut line is related with an intense influence of lateral forces on the tool flank surface [30]. As in the case of blanking, similarly, during the shearing process: Clearance affects the wear of tools [31]. Cho in his paper [31] described the influence of the direction of inclination of the DP980 sheet in relation to the punch, on the quality of the intersection surface. He characterized the failure mechanism of blanking punches in the case of using different directions of the sheet surface in relation to the punch surface. In order to reduce the defects of the intersection surface some modifications of the shearing process can be done [32,33]. Supporting the material in shearing process, with 20% of tool clearance, reduces the burr on the sheet separation edge [32]. Mackensen and others [33], showed that the proper arrangement of the trimmed sheet in relation to the surface of the punch causes a reduction in the blanking total work. Tests for two different sheet-punch arrangements were made for materials used in the automotive industry (including high-strength steels).

**Figure 2:**
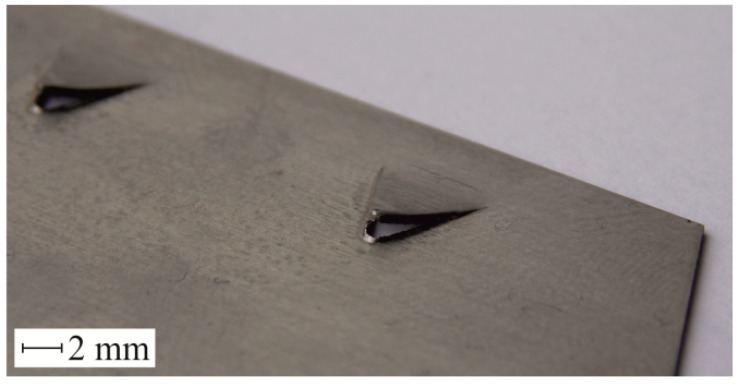
View of triangular hooks obtained by partial blanking.

In order to obtain cut out and bent fragments of sheets, punches with a sloping face are used [18]. In the case of blanking a triangular hook (Figure 2), there is a gradual deviation of the blanked material, in the direction of the punch movement. The burr on the edge of the sheet and the size of the deviation of the hook from the sheet surface, make it difficult to insert the hook into the other element during the assembly process. 

In the abovementioned manuscripts, the changes of the cut-part shape were not studied. The material area at the intersection surface, the quality of the intersection surface, and the burr were described as important issues. The partial blanking of parts of thin and very hard sheet material is not well researched and described.

The paper presents the results of research on the influence of different clearance, during partial blanking with a sloping face, on the geometrical quality of the hook. The influence of clearance on the change of burr height and the partially bended material, made of 55 HRC hardness steel sheet, was discussed.

## 2. Materials and Methods 

### 2.1. The Blanking Experiment

The hooks were cut in a sheet with a thickness of *t* = 0.5 mm. The sheet was made of C45 (1.0503) non-alloy carbon steel for thermal improvement [34]. Table 1 shows the chemical composition and Table 2 shows the mechanical properties after heat treatment.

**Figure 3:**
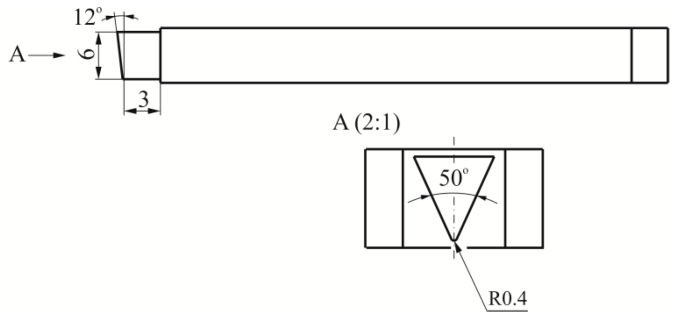
The basic geometry of the punch blanking tool.

**Figure 4:**
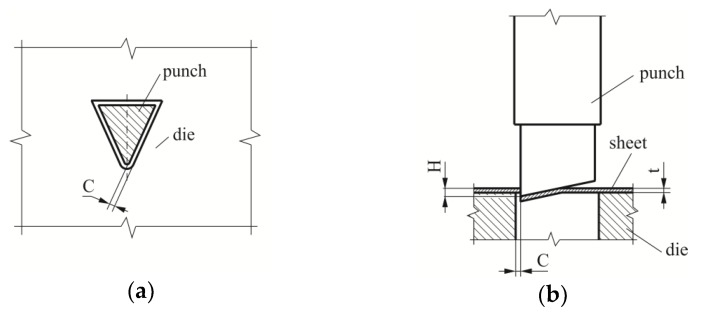
Characteristic elements of the tool and material arrangement: (**a**) Outline of the die hole and punch; (**b**) clearance (*C*) and blanking depth (*H*) of the punch in the sheet.

The blanking process was carried out with a punch with a 12° sloped face. The most important dimensions of the blanking punch are presented in Figure 3. During the tests, the punch movement was 50 strokes/min. The tests were carried out for three values of punching clearance (clearance (*C*)/sheet thickness (*t*)): 5%, 10%, 15% (Figure 4a). The stroke of the punch was set so as to obtain a penetration depth *H* = 1.2 mm (Figure 4b).

Sheet metal blanking with high strength properties requires, for punches, the selection of a material with good strength and relatively high resistance to abrasive and adhesive wear. In the case of short series of blanked products, the economics of the material used for the tools is important. The punch was made of K340 Isodur tool steel from Böhler Edelstahl Company (Düsseldorf, Germany). Steel with a content of ~8% chromium is produced in the technology of electroslag remelting process (ESR) [35]. This steel is characterized by, among other things, high adhesive resistance to wear and compressive strength. The basic alloying additions are presented in Table 3. Thanks to the micro-addition of aluminum, the oxide passivation system is improved, where passivation of the surface takes place. After passivation, this layer reduces the tendency of particles of punched material adhesion to the cutting surface of a punch. The hardness of the punch after heat treatment was 62 HRC.

### 2.2. Measurement of the Hook Profile and the Size of the Burr

**Figure 5:**
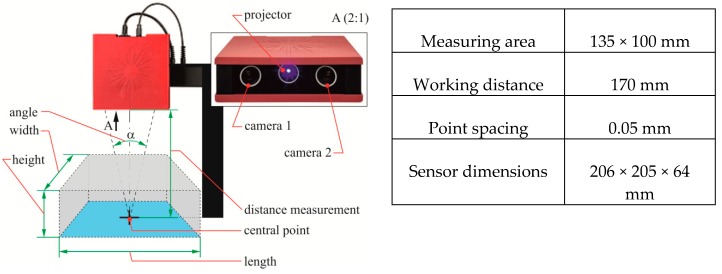
Idea of the AtosCore 135 measurement system and main camera parameters.

The measurement of the hook profile radius was determined using the non-contact method by using the GOM Atos optical scanning and measuring system. The Atos Core 3D scanner is a system of stereoscopic cameras, which work by triangulation (i.e., calculation of the intersection of a specific plane with a ray in three-dimensional space). The scanner projects a system of bands on the inspected surface of the piece. The projected bands are recorded by the two stereoscopic cameras (Figure 5), providing a phase-shift image from the sine distribution of intensity on the camera detectors.

**Figure 6:**
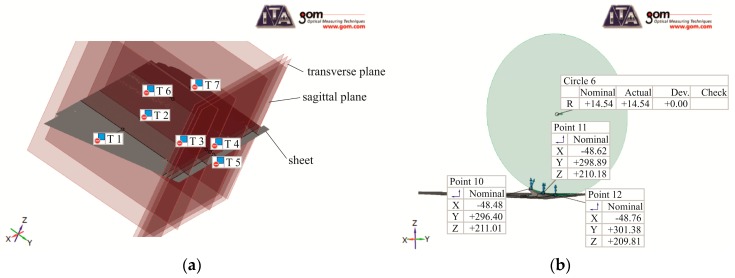
(**a**) Example of positioning base surfaces in the measuring system and (**b**) the principle of bending radius measurement.

**Figure 7:**
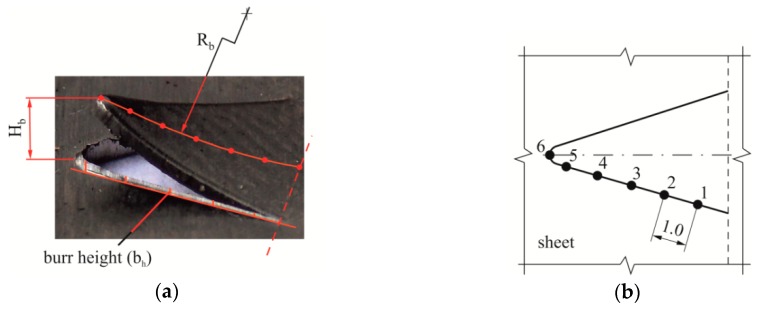
Burr characteristic measurement points (*b_h_*) and measurements of *R_b_* and *H_b_*: (**a**) Location of base points to determine the bending radius of the hook and (**b**) read points of the burr values.

In each case of the measurement, first a scan of the sheet with the punch was performed. The samples were placed in the same position relatively to the scanner. The obtained data allowed the remodeling of the sheet surface with a punched hook. After establishing the appropriate longitudinal and transverse surfaces (Figure 6a), the measuring line with points was determined (Figure 7a). The characteristic points were defined based on the length of the linear hole section in the hook axis. On their basis, the radius of the hook (*R_b_*) was determined. An example of defining points in the program and the circle described on them is shown in Figure 6b (the remaining points are hidden for better readability of the measurement principle). The size of the actual deviation (*H_b_*) of the hook from the main sheet was also measured (Figure 7a). The measurements of *H_b_* and *R_b_* were made for five samples.

**Figure 8:**
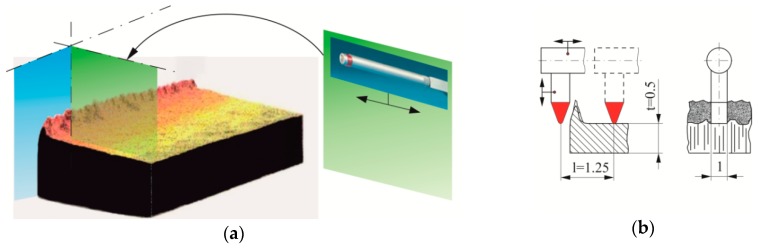
The idea of burr measurement: (**a**) System of base surfaces and (**b**) measurement scheme.

With the increase of the punch wear surfaces, the burr height changes [13,14,15,16,17]. By indirect method, through measuring the height of burrs on the elements, it is possible to determine which exploitation phase the punches are in [12]. The measurement of the burr on the edge of the hook was made on a stand, with a table adjusted by a micrometer screw, and using a TAYLOR HOBSON Surtronic 3+ profilographometer. The measurement accuracy was 0.5 m. Measurements on the selected section at the measuring points (Figure 7b) were made for the same places on five samples. The graphs show the average value from the measurements on five samples. The principle of determining the surfaces is shown in Figure 8a, and the scheme of burr measurement in Figure 8b.

After performing a hook scan with the ATOS system, the samples were cut using wire electrical discharge machining. The cut hooks were placed on the stand with a digital camera, where macro photographs were taken. To measure the burr, the samples were prepared in such a way that the bent hook was cut off. In this way, an easy access to the intersection edge on which the burr height was measured was obtained.

## 3. Results and Discussion

### 3.1. Bend of the Hook

**Figure 9:**
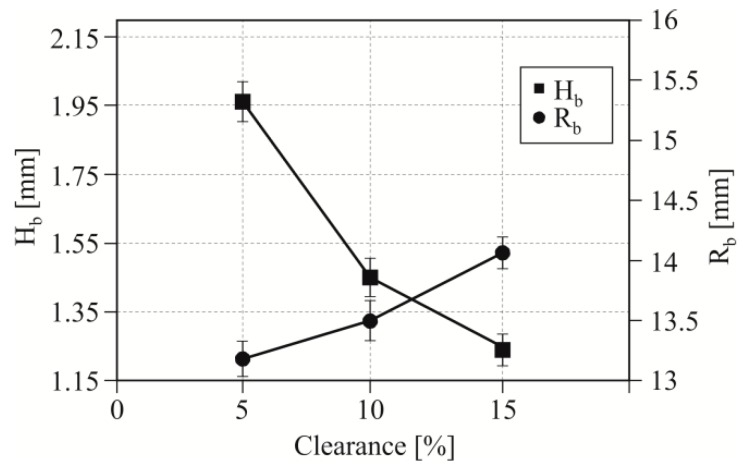
The influence of the blanking clearance (*C*) on the radius *R_b_* and the hook *H_b_* deviation after the first punching strokes.

In the initial phase of blanking, the punch deforms the material at the surface contact. When the punch pierces the sheet, the pressure phase ends for a part of the punch surface. Further movement of the punch results in a material cut and bending of the hook. For a 5% blanking clearance, the initial value of the deflection radius is smaller than when blanking with a clearance of 10% and 15% (Figure 9). Increasing the clearance from 5% to 10% caused the decrease of the deflection (*H_b_*) to approximately 21.4%. A further increase in clearance by 5%–15% resulted in a reduction in deflection value. The deflection (at *C* = 15%) decreased compared to that obtained at 5% clearance by 29.4%. The described dependences and changes in *H_b_* and *R_b_* values were observed for blanking process with a sharp punch.

During the tests, the cutting edge and the surfaces of the punch were worn out. During the blanking and measurement tests, the biggest differences in changes in *H_b_* and *R_b_* values were observed for the blanking clearance of 5% and 15%. Therefore, the further analysis presents the results of changing the geometry of the hook in the range of up to 60,000 cuts for 5% and 15% clearance.

**Figure 10:**
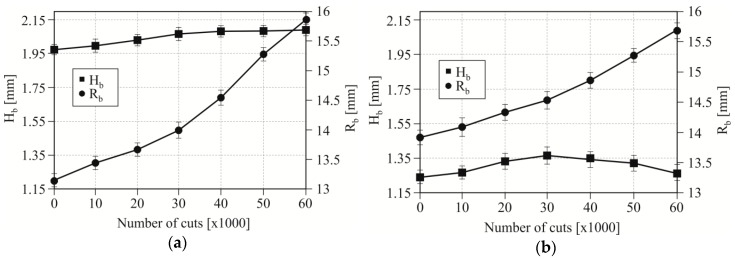
The influence of changes in *H_b_* and *R_b_* values depending on the number of cuts; for blanking clearance: (**a**) 5% and (**b**) 15%.

During punching with a clearance of 5%, the bend radius (*R_b_*) increased as the number of cuts made by the punch increased (Figure 10a). A significant increase in the radius value was observed since 30,000 cuts had been achieved. A further increase in the number of hook cuts caused a more intensive increase in the radius of the hook’s deflection. After about 30,000 cuts, the *H_b_* value began to grow to a lesser extent, up to around 40,000 cuts. Further, as the number of punch cuts increased, the radius of the hook deflection did not increase. On the other hand, the *R_b_* has increased quite significantly from the first cuts to 60,000 cuts. On the curve of the change in the size of *R_b_*, there can be observed an increase after reaching about 30,000 cuts. Blanking with a clearance of 5% did not cause a significant change in *H_b_* for the examined range of cuts.

In the case of blanking hooks with 15% clearance (Figure 10b), both the *R_b_* radius and the *H_b_* bend of the hook increased as the number of cuts increased to a similar one as at clearance of 5%. The size of the deflection (*H_b_*) of the hook until the excision of about 30,000 cuts increased and then began to decrease. The change of punching clearance from 5% to 15% indicated that after the first cuts *R_b_* values were higher by about 15.4% and *H_b_* deflections were higher by 16.6%. With 60,000 cuts, the differences in the change in value were different: *R_b_* was less by about 1% and *H_b_* was larger by about 50%. The 1% value change can be considered that the value was in the margin of error. To a greater extent, the clearance had an influence on the change of the *R_b_* bend hook. In spite of this, it can be concluded that the change in clearance, and therefore wear, had significantly changed the geometry of the hook during the blanking process with a use of a sloping-face punch. 

**Figure 11:**
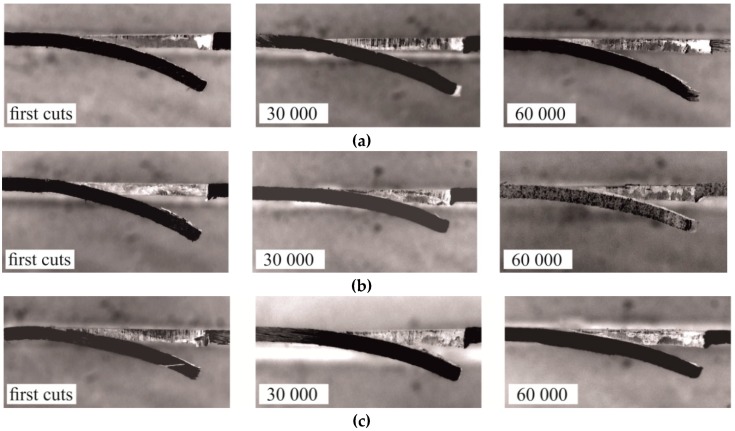
Cross-section of the hook for a particular number of punch cuts: (**a**) *C* = 5%; (**b**) *C* = 10%; and (**c**) *C* = 15%.

Figure 11 shows macro photographs of exemplary sections of a cut-out hook made with the use of a sloping-face punch. In the case of a 5% clearance, as the number of cuts increased, the deviation of the hook increased and the hook radius decreased (for 60,000 cuts) (Figure 11a). The intensive wear of the punch caused an increase in the clearance between the blanking edges of the tools. The progressive wear of the punch influenced the change in the geometry of the hook and the size of the clearance. The size of the burr on the edge of the sheet also was changing during the blanking process. As the number of cuts made by the punch increased (*C* = 5%), the burr on the edge of the sheet also increased. Increasing the clearance by 10%, i.e., to the value of 15%, resulted in the hook springing. After the first cut with a 15% clearance, the bend radius was greater than for the hook cut with a clearance of 5%. The increase in clearance also caused a significant increase in the size of the burr. The value of the hook deflection was the highest after the first cut for 10% blanking clearance (Figure 11b). As the number of cuts made by the punch increased, its wear increased, which had an influence on the decrease of the hook deflection value. The largest deflection of the hook (*C* = 10%) could be due to the increase in the influence of the moment of reaction forces of the cut material. However, insufficient clearance caused incomplete springing of the material, as in the case of punching with a clearance of 15% (Figure 11c). The corresponding values of parameters *H_b_* and *R_b_* for the element, first blanking 30,000 cuts and 60,000 cuts, are presented in Table 4. The hardness of tools and clearance between blanking edges [9] has an influence on the quality of the punched elements. Sheet metal blanking with low clearance causes more intensive wear of the blanking edge of punches [6,7,8].

### 3.2. Burr Height

**Figure 12:**
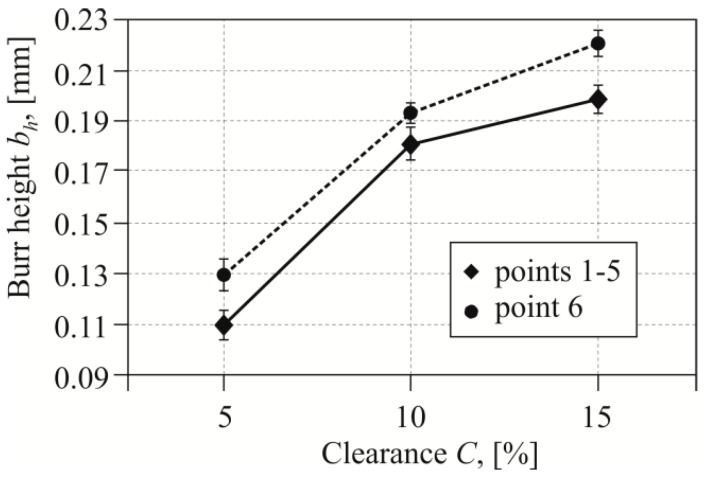
Effect of clearance on the size of the burr (after the first cuts with a punch).

When blanking carbon sheets with high hardness, as the number of cuts increased, a significant increase in burr height was observed [11]. Blanking with too low a clearance caused faster wear of the blanking edge. The roundness of the blanking edge changed the stress distribution in the tool edges contact area. Increasing the clearance from 5% to 10% resulted in a burr increase of the average value of 63% (Figure 12—solid line). Increasing the clearance by another 5% resulted in another increase of the burr by 12% of the value obtained as for a 10% clearance. The presented values and their changes refer to the average value of the burr from five points (as in Figure 7b).

**Figure 13:**
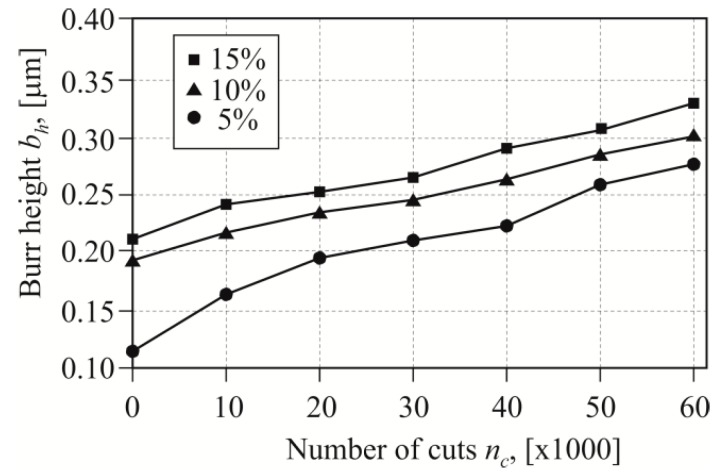
Influence of the number of cuts on the size of the burr at the fifth measurement point (Figure 7b).

During the tests, it was observed that on the rounded edges (point 6 in Figure 7b) the burr is slightly larger. The largest difference between the average value of the burr on the edge (for points 1 to 5) and the one measured at the rounding of the edge (point 6) was obtained for the 15% clearance (Figure 12—dashed line). The smallest difference between the values of the burr was observed in the case of punching with a clearance of 10%. The higher burr values at the edge of the semicircular metal sheet were caused by the non-linear movement of the blanking edge at this location. In point 6, the rounding of the edges was affected by the deformations caused by blanking with linear edges. It was a relatively small area, located near the top of the punch with a radius of 0.4 mm (Figure 3 and Figure 4). The hooks were characterized by high elasticity, so that they did not plastically deform in the subsequent assembly process. The hook blanking tests were carried out for carbon steel sheet hardened to 55 HRC. In the case of a 5% blanking clearance the largest increase in burr, at the tested point on the edge of the hole, occurred up to 20,000 cuts (Figure 13). Up to 30,000−40,000 cuts, the burr increase stabilized. Blanking with a punch in the range from 40- to 60 thousand strokes had already caused a significant increase in the burr. In the last cut range, the process of punch wear was more intense, hence, the burr on the edges of the sheet increased. Blanking the hook with a clearance of 10% resulted in a less intense burr increase, but with a higher value compared to blanking with a clearance of 5%. The curve in the range of up to 30,000 cuts was gentler rising, and the burr had increased more intensively. In the case of blanking with a clearance of 15%, a more intense increase of the burr was observed up to a value of about 30,000 cuts. For all clearance values, the cut range from 0 to 30,000−40,000 is the range of intense burr increase at the edges of the sheet. The punch had a sloping face, the contact (path of friction) of the separated material with the side surface of the punch was not the same (along the blanking edge). Therefore, we decided to analyze the value of the burr growth, for the two extreme clearance values of 5% and 15%, depending on the number of cuts.

**Figure 14:**
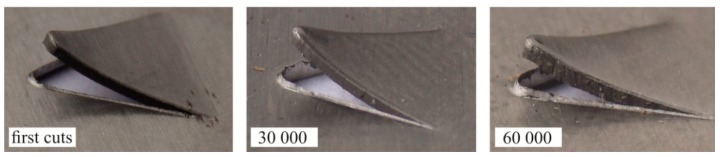
The burr on the edge of the sheet for exemplary hooks cut out with a clearance of 5%.

**Figure 15:**
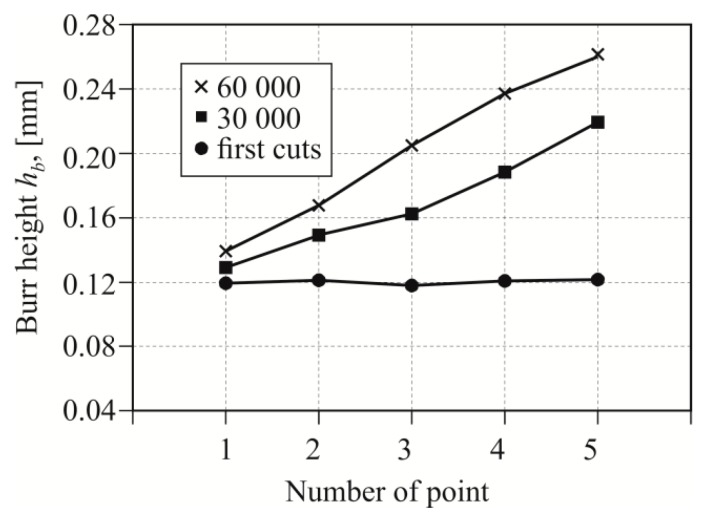
Burr height values measured along the blanking edge for five measuring points (*C* = 5%).

Furthermore, during the tests, it was also observed that the burr along the straight edge was not the same (Figure 14). After the first punch cuts, the burr along the straight edge was the same. After further cuts, the irregularity of the burr increased. The higher the punch wear, the larger the variation of the burr on the straight edge. At the pattern roundness, the height of the burr changed in a slightly different way. When blanking material, the face and cutting edge were more loaded (material impact). Cyclical pressure loading close to the cutting edge caused the punch fatigue wear. Thus, the burr was not proportional to the burr on the straight line. The examples of the measurement results, in the characteristic points on the sheet edge, for the three values of the number of punch cuts (*C* = 5%) are presented in Figure 15.

**Figure 16:**
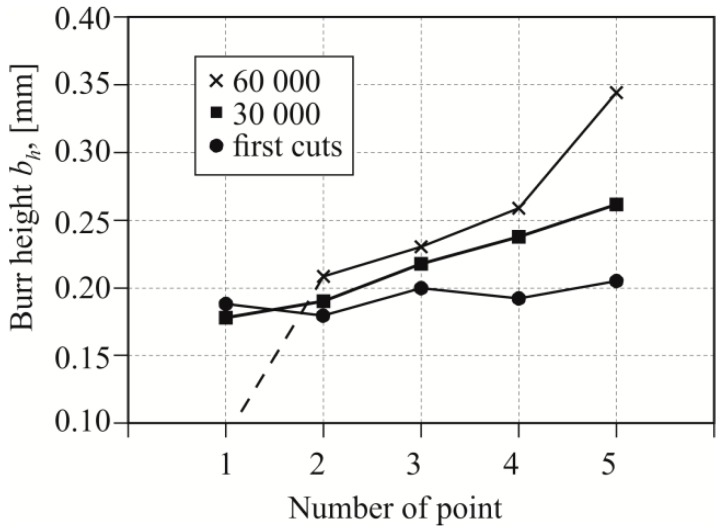
Burr height values measured along the blanking edge for five measuring points (*C* = 15%).

**Figure 17:**
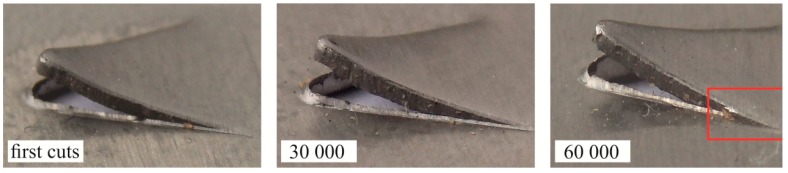
Burr (*b_h_*) on the edge of the sheet for hook cuts out with a clearance of 15%.

**Figure 18:**
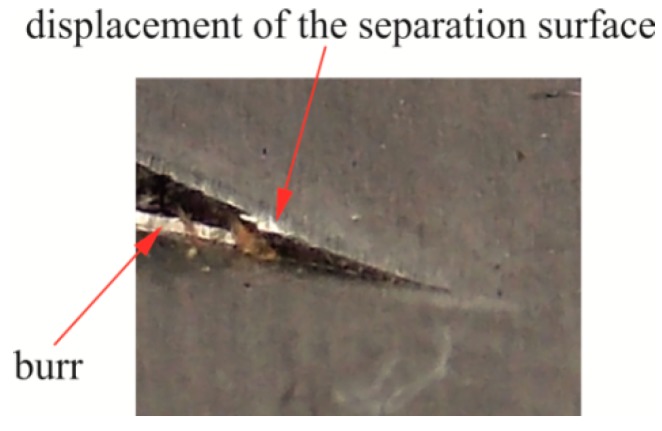
Zoom area (in Figure 17) near the separation of the material of the hook deflection.

In the case of blanking with 15% clearance (Figure 16), the burr edge on the edge was much higher than for the 5% clearance (Figure 15). The greater the friction path with a single stroke, the more the burr on the edges of the sheet increased. Within the value of around 30,000 cuts, the burr proportionally expanded along the linear edge of the sheet. From about 30,000 cuts with a clearance of 15% (Figure 13 and Figure 16), the burr size change was not proportional. After 60,000 punch cuts, the variation of wear of the punch cutting surfaces was significant. Photographs of hook samples blanked with a clearance of 15% are shown in Figure 17. The variation of wear was observed to such an extent that the gap between the blanking edges had changed, and the material separation surface displacement was observed (Figure 17—detail, Figure 18). The burr on the part edge practically was not obtained (Figure 18).

### 3.3. The Mechanism of Wear and Damage to the Punch

**Figure 19:**
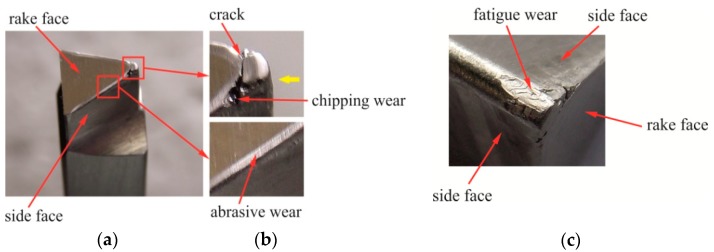
Tool after blanking about 63,000 elements with 5% clearance: (**a**) the principal blanking-punch elements; (**b**) cutting edge wear; (**c**) fatigue wear.

**Figure 20:**
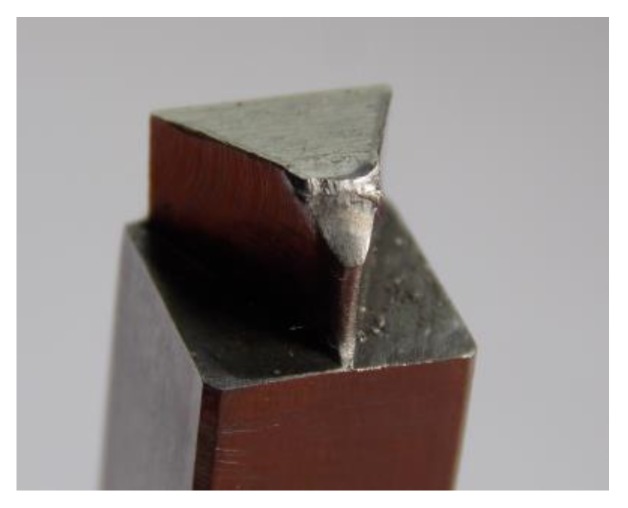
A punch with a sloping frontal surface after damage and punch material separation (*C* = 5%, 65,000 cuts).

As previously presented, the burr was not the same on the edges of the sheets, hence it was decided to make a brief analysis of the mechanism of wear and damage to the punches. In the blanking process with 5% clearance, after exceeding 60,000 cuts, an incremental increase in the value of the burr size was observed. For 63,000 cuts, the punch had microcracks and bigger wear of the blanking edges (Figure 19). The tests were discontinued and the punch was replaced. The next punch was damaged after about 65,000 cuts (Figure 20). It was observed that up to the limit of 60,000 cuts, the burr on the edge did not increase incrementally. Hence, for comparative purposes, the deflection (*H_b_*) and bending radius (*R_b_*) analysis of the hook and the size of the burr (*b_h_*) of their values have been prepared for 60,000 cuts.

The blanking of hooks in the hardened steel sheets (55 HRC) caused the surfaces of flank and tool face of the punch (Figure 19a) to be abrasively worn (Figure 19b—lower) and fatigue worn (Figure 19b—upper and Figure 19c). Near the top of the punch (Figure 19b—upper), fatigue fractures and material damages were visible. The abrasive wear along the blanking edge occurred on the leading (face) surface. The abrasive wear on the face of the punch near the top had a slightly larger size. The contact area of the punch with the material was initially larger. Only after material cutting out, the hook was gradually bent. During the gradual blanking, there was a micro-slip between the surface of the bending material of the hook and the face of the punch.

**Figure 21:**
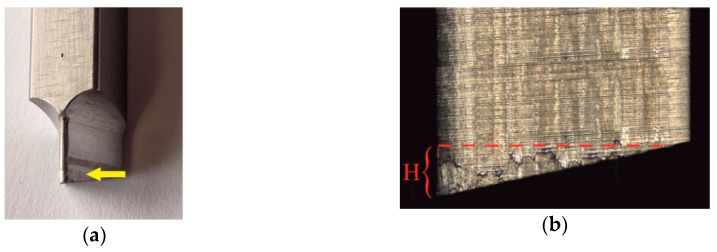
Tool after blanking 60,000 elements with 15% clearance: (**a**) the blanking part of the punch; (**b**) flank wear.

On the flank surface of the punch with the sloping face (Figure 21a), the wear was not the same (Figure 21b). This is due to the fact that the length of the friction path was different: The largest was at the top of the punch. The surface near the cutting edge must pierce the material, the material is cut out by the punch’s cutting edges. The deflection of the hook was obtained by gradual material blanking, and bending the punch stroke to the depth *H* (Figure 4b) separates the material along the blanking edge of the punch. The face of the punch was inclined at an angle (α). The area of pressure between the sheet and the punch moves along the edge. The bending of the cut part, in the transverse plane, caused the pressure to be concentrated near the blanking edge at a certain width. As a result of blanking a certain number of hooks on the surface of the punch, damage occurred (Figure 19b).

**Figure 22:**
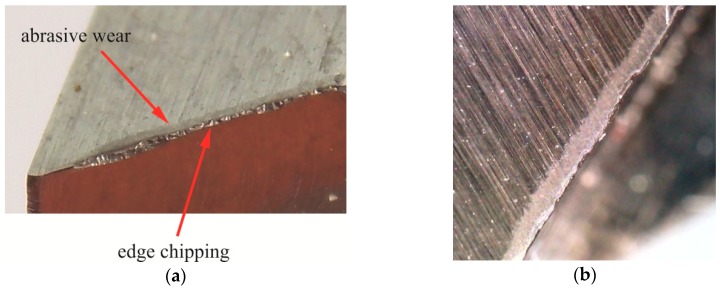
The punch after blanking out about 60,000 hooks with a clearance of 10%: (**a**) A fragment of the blanking edge of the punch with visible chippings and (**b**) abrasive wear on the face close to the blanking edge.

Punching with an intermediate clearance between 5% and 15%, i.e., *C* = 10%, caused the wear of the blanking edge of the punch to a greater extent than in the case of the two remaining clearances (Figure 22a). Blanking the hooks with the clearance *C* = 10% in a C45 steel sheet, with hardness 55 HRC, caused chipping of the blanking edge and abrasive wear of the face of the K340 steel punch. A band of abrasive material wear near the chipped edge of the punch is visible on the punch face (Figure 22b).

## 4. Conclusions

This experimental research has shown how the determined value of clearance, in the process of partial blanking of a C45 sheet with a hardness of 55 HRC, affects the size of burrs on the edges of the sheet. In addition, the obtained results of the *H_b_* deviation of the hook and the size of its radius, *R_b_*, allowed to assess how the wear of the punch influences the course of changes in the geometric values of the cut element. Based on the results achieved from the research, the following conclusions can be formulated. 

1. In the case of K340 material of the punch, it was possible to cut the hooks to a range of approximately 60,000 cuts. Longer exploitation caused high fatigue of cold-work tool steel material. The size of the blanking clearance very clearly affected the intensity of wear of the blanking edge of the punch and the amount of deflection of the hooks.

2. During the tests after the first cuts with a punch, only for the clearance of 15% the value of the hook deviation *H_b_* = 1.2 mm was obtained. In the case of the other two partial blanking experiments with 5% and 10% clearance, the *H_b_* values were higher than assumed. For a 5% clearance, the *H_b_* value obtained after the first cut was 62.5% higher, while for *C* = 10% the deflection was 18.3% higher. As the number of cuts increased, the wear of the punch caused significant changes in the deflection value.

3. The use of a punch with a sloping face for partial blanking of the hooks in the C45 sheet, resulted in irregular wear of the punch. Blanking and bending an element with an open separation line results in irregular wear of the blanking edges. As the number of cuts made by the punch increased, the size of the burr was diversified.

4. The value of the burr for the three blanking clearance values was different. The lowest initial burr values were obtained for a 5% clearance, and the biggest one for 15% clearance. However, the largest increase in burr values was observed during blanking with a clearance of 5%. After 60,000 cuts, with 5% clearance, on the linear edge the burr value increased by 133%.

## Figures and Tables

**Table 1:** Chemical composition (average), [%].

C	Mn	P	Cr	Si	S	Ni	Mo	Fe
0.48	0.73	0.011	0.09	0.35	0.01	0.02	0.002	other

**Table 2:** Mechanical properties of hardened steel C45.

Yield Strength Re [MPa]	Tensile Strength Rm [MPa]	Elongation A5 [%]	Hardness HRC
335	2285	30	55

**Table 3:** Chemical composition of the cold-work tool steel K340 (in weight percent).

C	Si	Mn	Cr	Mo	V	Other Micro-Alloying
1.10	0.90	0.40	8.30	2.10	0.50	Al, Nb

**Table 4:** Size *R_b_* and *H_b_* depending on the clearance and the number of cuts made by the punch.

Number of Cuts	Clearance, *C*			Clearance, *C*		
	5%	10%	15%	5%	10%	15%
	*R_b_* [mm]			*H_b_* [mm]		
First cuts	13.15	13.52	14.05	1.96	1.42	1.21
30,000	13.92	14.01	14.61	2.11	1.52	1.36
60,000	15.75	15.77	15.71	2.12	1.67	1.28
